# Plasma homocysteine levels and genetic polymorphisms in folate metablism are associated with breast cancer risk in chinese women

**DOI:** 10.1186/1897-4287-12-2

**Published:** 2014-02-21

**Authors:** Xiayu Wu, Tianning Zou, Neng Cao, Juan Ni, Weijiang Xu, Tao Zhou, Xu Wang

**Affiliations:** 1 School of Life Sciences, The Engineering Research Center of Sustainable Development and Utilization of Biomass Energy, Ministry of Education, Yunnan Normal University, Kunming, Yunnan 650500, China; 2 Third Affiliated hospital of Kunming Medical College, Kunming, Yunnan 650101, China

**Keywords:** Folate, SHMT, MS, MTRR, CBS, Hcy, Breast cancer, Risk

## Abstract

**Background:**

Folate plays a pivotal role in DNA synthesis, repair, methylation and homocysteine (Hcy) metabolism. Therefore, alterations in the folate-mediated one-carbon metabolism may lead to abnormal methylation proliferation, increases of tumor/neoplasia and vein thrombosis/cardiovascular risk. The serine hydroxymethyhransferase (SHMT), methionine synthase (MS), methionine synthase reductase (MTRR) and cystathionine beta synthase (CBS) regulate key reactions in the folate and Hcy metabolism. Therefore, we investigated whether the genetic variants of the *SHMT, MS, MTRR* and *CBS g*ene can affect plasma Hcy levels and are associated with breast cancer risk.

**Methods:**

Genotyping was performed by PCR-RFLP method. Plasma Hcy levels were measured by the fluorescence polarization immunoassay on samples of 96 cases and 85 controls.

**Results:**

(a) The *SHMT 1420 T, MS 2756G, MTRR 66G* allele frequency distribution showed significant difference between case and controls (p < 0.01 ~ 0.05). (b) The concentration of plasma Hcy levels of *SHMT 1420TT* was significantly lower than that of the wild type, while the plasma Hcy levels of *MS 2756GG*, *CBS 699TT/1080TT* significantly higher than that of the wild type both in case and controls. The plasma Hcy levels of *MTRR 66GG* was significantly higher than that of wild type in cases. The plasma Hcy levels of the same genotype in cases were significantly higher than those of controls except *SHMT 1420CC, MS 2756AA, MTRR 66GG*; (c) Multivariate Logistic regression analysis showed that *SHMT C1420T* (OR = 0.527, 95% CI = 0.55 ~ 1.24), *MS A2756G* (OR = 2.32, 95% CI = 0.29 ~ 0.82), *MTRR A66G* (OR = 1.84, 95% CI = 0.25 ~ 1.66) polymorphism is significantly associated with breast cancer risk. And elevated plasma Hcy levels were significantly linked to increased risk of breast cancer (adjusted OR = 4.45, 95% CI = 1.89-6.24 for the highest tertile as compared with the lowest tertile).

**Conclusions:**

The current study results seem to suggest a possibility that S*HMT C1420T* mutation may be negatively correlated with breast cancer susceptibility; while *MS A2756G* and *MTRR A66G* mutation may be positively associated with breast cancer risk. *SHMT C1420T, MS A2756G, MTRR A66G, CBS C1080T, CBS C699T* locus mutation may be factors affecting plasma levels of Hcy. The plasma Hcy levels could be metabolic risk factor for breast cancer risk to a certain extent.

## Background

About 1.2 million women suffer from breast cancer in the world every year, and China is one of countries with most rapid increase in incidence of the disease, which is already up to 200,000 persons per year, *c.* 40,000 Chinese die of the disease [1]. In this deadly disease, the mortality rate approximately equals the incidence rate. The etiology of breast cancer is poorly understood [2]. High-risk populations include those with a family history of breast cancer. Several familial syndromes with known genetic defects have been implicated, but they account for <6% of the total cases [3]. The risk factors most consistently established by epidemiologic studies are age, cigarette smoking, alcoholism and estrogen level changing or hormone receptor status [2,4,5]. Two prospective studies have since shown that the increased risk of breast cancer associated with low folate and high alcohol intake is limited to estrogen receptor-negative breast cancer [4,5].

Folate is critical to one-carbon metabolism, acting as a coenzyme in facilitated novo deoxynucleoside triphosphate synthesis and to provide methyl groups required for intracellular methylation reactions. Folate has generally been thought to be safe and protective against anemia, atherosclerosis, neural tube defects, adverse pregnancy outcomes, neuropsychiatric disorders and cancer [6-8]. There is evidence that it can promote the growth of preneoplastic lesions and recent work has emphasized the importance of timing and dose of folate in the carcinogenesis process [9,10]. A large number of epidemiological studies point to dietary folate, which is plentiful in vegetables and fruits, has been associated with reduced risk of several cancers [11-14]. Several large prospective epidemiological studies have suggested an importance of folate in reducing breast cancer risk, particularly among women who regularly consume alcohol [15,16].

Homocysteine (Hcy) is a non-protein-forming, sulfur containing amino acid, formed exclusively by demethylation of methionine and degraded by remethylation or transsulfuration [17]. Hcy plasma levels are influenced by genetic polymorphisms of key enzymes involved in folate and methionine metabolism. Recent studies have shown that low folate status, with hyperhomocysteinemia as a consequence, is associated with oncogenic risk in patients with inflammatory bowel disease [18], probably due to hypomethylation [19]. Aberrant methylation of DNA is frequently found in tumor cells [20]. Global hypomethylation can result in chromosome instability [21], whereas region-specific hypermethylation has been associated with the inactivation of tumor suppressor genes [22].

Hcy is an immediate precursor for the biosynthesis of influences S-adenosylhomocysteine (SAH) and hyperhomocysteinemia results in impaired balance between methionine, S-adenosylmethionine (SAM), and SAH. This imbalance may lead to changes in methyl-donor substrate levels and consequently to a modulation of DNA methylation pattern [23]. Hcy is also a potent inhibitor of DNA hydroxymethyltransferase being SAH inhibits DNA hydroxymethyltransferase. Additional Hcy is converted to SAH pathway may involve folate-dependent reactions leading to changes in the availability of nucleotides, such as thymidylate, for DNA synthesis and repair [24]. This mechanistic hypothesis suggests that hyperhomocysteinemia exerts its pathogenic effects largely through metabolic accumulation of intracellular SAH, a strong non-competitive inhibitor of the catechol-O-methyltransferase (COMT)-mediated methylation metabolism of endogenous and exogenous catechols (including 2-OH-E2 and 4-OH-E2) [19,20]. The oxidation of endogenous catechols generates large amounts of chemically reactive products, (such as quinone/semiquinone intermediates) that are highly toxic to surrounding cells [21]. A strong inhibition of the methylation metabolism of 2-OH-E2 would significantly decrease the formation of 2-MeO-E2 (an endogenous anticarcinogenic metabolite of E2) and an inhibition of the methylation of 4-OH-E2 would lead to accumulation of this hormonally active and strongly procarcinogenic estrogen metabolite [22]. Both of these effects resulting from the inhibition of the methylation metabolism of catechol estrogens would facilitate the development of estrogen-dependent carcinogenesis in the target organs.

For all these reasons, hyperhomocysteinemia has been regarded as a risk factor for cancer and Hcy levels have been proposed as a new tumor marker since they not only accurately reflect the proliferation rates of tumor cells but also respond to tumor cell death [25].

**Figure 1:**
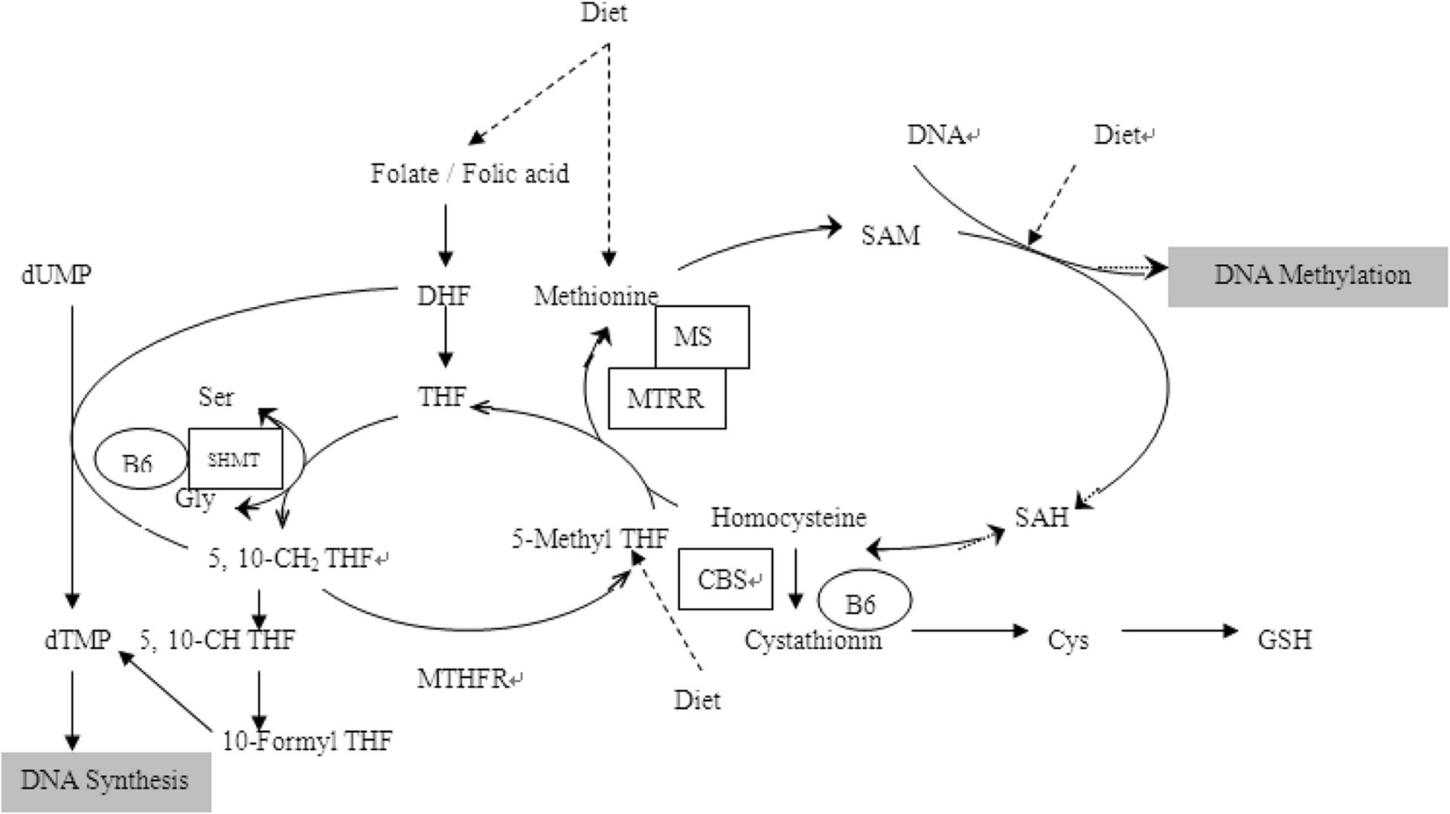
**Overview of the folate-methionine metabolic pathway investigated in this study.** THF, tetrahydrofolate; DHF, dihydrofolate; 5, 10-CH_2_ THF, 5,10-methylenetetrahydrofolate; 5-Methyl THF, 5-methyl tetrahydrofolate; dUMP deoxyuridine monophoshate; dTMP, deoxythymidine monophoshate; SAM, S-adenosylmethionine; SAH, S-adenosylhomocysteine; SHMT, serine hydroxymethyhransferase; MS, methionine synthase; MTRR, methionine synthase reductase; CBS, cystathionine beta synthase; B6, vitamin B6; Ser, serine and Gly, glycine.

Serine hydroxymethyltransferase (SHMT), methionine synthase (MS), methionine synthase reductase (MTRR) and cystathionine beta synthase (CBS) are genes that encode enzymes involved in the folate and Hcy metabolic pathway, which are critical in the DNA synthesis, methylation process and Hcy metabolism (Figure 1) [26]. Studies have suggested that polymorphisms in these genes may be related to plasma Hcy levels increasing and cancer development [27-29], but the study on interaction between plasma Hcy levels, above-mentioned genetic polymorphisms in folate pathway and breast Cancer susceptibility is still lacking. Thus the aim of this study was to analyze the possible associations between Hcy concentration in serum, polymorphisms of enzymes involved in folate/methionine metabolism and breast cancer susceptibility. This knowledge is important for enhancing our understanding and improving the effectiveness of our public health recommendations Figure 1.

## Methods

### Case and control selection

Eligible patients with breast cancer were randomly selected from 2010 January to 2011 April in the Third Affiliated Hospital of Kunming Medical College (the tumor hospital of Yunnan province), Yunnan, China. Based on the hospital chart number, cases involved 96 women consecutively selected from subjects with a first confirmed histopathologic diagnosis of breast carcinoma in the age range of 30–77 years. The each types of cancers including infiltrating ductal carcinoma (15 individuals), papillary carcinoma (11 individuals), invasive ductal intraductal carcinoma (40 individuals), smooth cribriform carcinoma (10 individuals), medullary carcinoma (8 individuals), fibrosarcoma (3 individuals), non invasive ductal intraductal carcinoma (4 individuals) and accessory breast malignant tumor (5 individuals). Eighty-five Control subjects comprising individuals without a history of cancer in the age range of 18–76 years were simultaneously recruited form the health examination clinics of same hospital during the same study period. Eventually, the controls were individually matched for age (±3 years) and menopausal status (premenopause or postmenopause) to cases with a 1: 1 case–control ratio.

### Collection of questionnaire data and blood specimens

Approval for the present study was obtained from the National Natural Sciences Foundation of China (NSFC) and the Yunnan Scientific and Technological Committee. Once case patients and control subjects agree to participate, written informed consent was obtained from all subjects. Pathology tests were performed and the number of active manifestations recorded for each patient. The pathology tests included morphological pathological examination of breast, nipple discharge cytology, cytological smear, biopsy, endoscopic examination and so on. Data were collected on sociodemographic characteristics, menstrual and reproductive history, menopausal status, lifestyle behaviors and medical history as well as family history of breast and other cancers. More specifically, in this study, menopausal status was defined as last menstruation after one year free of menstrual cycle and no attempt was made to distinguish between women with artificial and those with natural menopause. Subjects taking vitamin supplements in the previous 6 months were excluded from the study.

### Measurement of plasma levels of Hcy

Plasma Hcy concentrations (ie., plasma total Hcy measured as the sum of all Hcy subfractions in plasma including free and protein-bound forms) were determined by fluorescence polarization immunoassay (FPIA) technique (Axis Biochemicals, ASA, Oslo, Norway). FPIA was run on an IMx analyzer (Abbott, Ill, USA). The 95% confidence interval of plasma Hcy level suggested by the manufacturer for healthy individuals is 4.45–12.42 *μ*mol/L. Five cc blood samples were drawn using a 25 gauge needle from a peripheral vein, avoiding haemolysis in the morning hours after an overnight fasting and 30 minutes of supine rest and collected into 10 mL empty evacuated tubes without EDTA, heparin, or clot activators. Samples were centrifuged at 1000 × g for 10 minutes. The plasma was separated in aliquots and immediately frozen and stored at −80°C within 60 minutes until use.

### Genotyping of the *SHMT C1420T, CBS C699T/C1080T, MS A2756G*, *MTRR A66G* polymorphisms determination

Genomic DNA was extracted from peripheral leukocytes isolated from acid-citrate-dextrose-anti-coagulated blood using a commercially available FlexiGen DNA isolation kit (Qiagen, Valencia CA).

The polymorphisms *MS A2756G* were determined by polymerase chain reaction (PCR) followed by restriction fragment length polymorphisms (RFLP) [30]. The amplified fragments of *MS A2756G* polymorphisms were digested with restriction enzymes, *Ha*e*III* (Beijing Biological Engineering Co. Ltd., China). *MTRR A66G* genotype was analyzed by PCR followed by RFLP as described by Scazzone et al. [28]. The amplified DNA fragment was digested with *NdeI* (Beijing Biological Engineering Co. Ltd., China). The polymorphisms *T1080C* and *C699T* in *CBS* gene were determined using PCR-RFLP based assays using restriction enzymes *BstUI* (Beijing Biological Engineering Co. Ltd., China) for *T1080C* and *RsaI* (Beijing Biological Engineering Co. Ltd., China) for *C699T*[31,32]. About 10% of the samples were reexamined by an investigator who had not attended the previous collection of data. There were no discrepancies in the results.To determine the *SHMT C1420T* polymorphism an allele discrimination method using fluorogenic 3′-minor groove binding (MGB) probes described by Skibola et al. was adapted [33]. The real-time PCR was performed in Rotorgene 2000 real-time cycler (Corbette Research, Mortlake, Australia). About 10% of the samples were parallel genotyped by real-time PCR and PCR-RFLP method using *Eam1104I* restriction enzyme (Fermentas Inc., Hanover, MD, USA). The discrepancy between the methods was below 1%. All products were electrophoresed at room temperature for 7 h at 300 volts on a 8% polyacrylamide gel (2% C) containing 5% glycerol and silver stained.

Genotypes of *MS A2756G*, *MTRR A66G*, *CBS C1080T/ C699T*, *SHMT C1420T* were defined as *2756AA/66AA/ 1080CC/699CC/1420CC* (ancestral homozygous) also reported in the literature as wild homozygous, *2756AG/66AG/1080CT/699CT/1420CT* (mutant heterozygous) and *2756GG/66GG/1080TT/699TT/1420TT* (mutant homozygous).

### Statistical analysis

Descriptive statistics were calculated to compare effectiveness of matching variables (age) and to assess breast cancer risk factor information. Crude odds ratios, t-tests and chi-square tests were examined to determine which variables were statistically significant associated with breast cancer risk. Covariates that did not change the effect estimate of fungicides on breast cancer risk by more than 10% were not included in the final logistic regression model. As cases and controls in this study were frequency matched, the analysis utilized unconditional logistic regression and included the matching variables. The results are given as odds ratio (OR) with 95% confidence interval (95% CI) were estimated using age-matched conditional logistic models adjusted for potential confounders. Trends in the OR (gene dosage effect) were calculated by assigning ordinal values to the genotypes. A non-parametric test for trend was used to look for differences in Hcy levels by genotype. For each polymorphism, deviation from Hardy–Weinberg equilibrium for the genotype distribution was evaluated in controls only, using an exact test. All genotypes were found to be in Hardy–Weinberg equilibrium. All analyses were performed using STATA 10.1 (College Park, TX) and the Statistical Package for Social Sciences (SPSS) version 15.

## Results and discussion

### Characteristics of case and controls

Table 1 showed the study population consisted of 96 breast cancer cases and 85 controls with a mean (±SD) age of 47.83 (±10.9) years and 45.22 (±11.8) years, respectively. There were no significant differences between cases and controls in terms of age at menarche (12.7 ± 1.2 years *vs.* 13.8 ± 1.4 years), age at first full-term pregnancy (28.4 ± 6.4 years *vs.* 25.9 ± 3.8 years), and age at menopause (50.7 ± 7.9 years *vs.* 52.5 ± 6.6 years). No significant differences were found between cases and controls in terms of the proportions of women with alcohol drinking (10.4% *vs.* 4.7%), post-menopausal women (28.1% *vs.* 24.7%). Smokers were no significantly more common in cases than in controls (14.6% *vs.* 0%).

**Table 1:** Characteristics of case and controls

	**Cases (n = 96) n (%)**	**Controls (n = 85) n (%)**	***p***
Age			
18–29	12(12.5)	9(17.6)	
30–39	25(26.0)	22(25.9)	
40–49	37(38.5)	37(43.5)	
50–59	14(14.6)	13(15.3)	
60–69	4(4.17)	3(3.53)	
70–79	4(4.17)	1(1.18)	
Mean age (SD)	47.83(±10.9)	45.22(±11.8)	0.37
Drinking habit			
Never	83(89.6)	81(95.3)	
Former^a^	6(6.25)	0(0)	
Current	4(4.17)	1(1.18)	
Moderate^b^	2(2.08)	1(1.18)	0.21
Heavy^c^	1(1.04)	0(0)	
Unknown	0(0)	3(3.53)	
Somking habit			
Never	76(79.2)	85(100)	<0.01
Former^a^	14(14.6)	0(0)	<0.01
Current (pack years)	6(6.25)	0(0)	
0–19	2(2.08)	0(0)	
≥20	3(3.12)	0(0)	
Unknown	1(1.04)	0	
BMI			
<18.5	9(9.38)	7(8.24)	
18.5–24.9	30(31.3)	26(30.6)	
≥25.0	53(55.2)	50(58.8)	
Unknown	4(4.17)	2(2.35)	0.87
Regular exercise			
Yes	62(64.6)	63(74.1)	
No	29(30.2)	21(21.9)	
Unknown	5(5.21)	1(1.04)	0.11
Family history of breast cancer			
Yes	0(0)	0(0)	
No	96(100)	85(100)	
Unknown	0(100)	0(100)	1.00
Menopausal status			
Premenopausal	69(71.9)	64(75.3)	
Postmenopausal	27(28.1)	21(24.7)	0.74
Age at menarche			
≤12	24(25)	19(22.4)	
13–14	66(68.8)	60(70.6)	
≥15	2(2.08)	44.70)	
Unknown	4(4.16)	2(2.35)	0.82
Age at menopause			
≤47	4(4.17)	2(2.35)	
48–52	13(13.5)	10(11.8)	
≥53	9(9.38)	7(8.24)	
Unknown	1(1.04)	2(2.35)	0.91
Others	2(2.08)	1(1.17)	0.21

SD, standard deviation; BMI, body mass index.

^a^Former smokers and drinkers were defined as subject who had quit smoking and drinking at at least 1 year previously.

^b^Moderate drinker <5 days per week.

^c^Heavy drinker **≥5** days per week.

### Effect of plasma Hcy levels on risk of breast cancer

Table 2 presents high plasma Hcy was statistically significantly associated with increased risk of breast cancer in the analysis controlling for age at enrollment and duration of fasting; the OR comparing the highest with the lowest tertile of plasma Hcy was 4.45 (95% CI = 1.89–6.24). To explore possible modifying effect on the association between plasma Hcy concentrations and breast cancer risk by menopausal status, Table 3 shows stratification by menopausal status in the association between plasma Hcy levels and breast cancer risk.

**Table 2:** Risk of breast cancer associated with plasma homocysteine (Hcy) concentration

**Variable**^**a,b**^	**Cases (n = 96) n (%)**	**Controls (n = 85) n (%)**	**OR**^**c **^**(95% CI**^**c**^**)**
Plasma total Hcy(μ mol/L)			
<7.26	16(16.7)	28(32.9)	1.00(reference)
7.26–11.56	11(11.4)	30(35.3)	0.64(0.23–1.12)
>11.56	69(71.9)	27(31.8)	4.45(1.89–6.24)

^a^The total number of cases and controls does not correspond because of missing data.

^b^Variables were categorized based on the tertile distribution among control subjects.

^c^OR, odd ratio; CI, confidence interval.

^c^Adjustment for age at enrollment and duration of fasting.

**Table 3:** Odds ratio (OR) and 95% confidence interval (CI) of breast cancer by the tertile distribution of plasma total homocysteine, with stratification according to menopausal status

**Stratified variable**^**a,b**^	**Plasma total Hcy (μ mol/L)**			
	**1st**	**2nd**	**3rd**	***p***
Menopausal status				
Pre-menopausal				<0.001
Cases/controls	8/20	4/19	57/25	
OR^c^ (95% CI^c^)	1.0(reference)	0.53(0.2-1.1)	5.7(2.7-8.8)	
Post-menopausal				<0.001
Cases/controls	8/8	7/11	12/2	
OR^c^ (95% CI^c^)	1.0(reference)	0.64(0.2-1.5)	6(3.1-8.2)	

^a^The total number of cases and controls does not correspond because of missing data.

^b^Variables were categorized based on the tertile distribution among control subjects.

^c^OR, odd ratio; CI, confidence interval.

^c^Adjustment for age at enrollment and duration of fasting.

### Analysis of gene polymorphisms and risk of breast cancer

**Figure 2:**
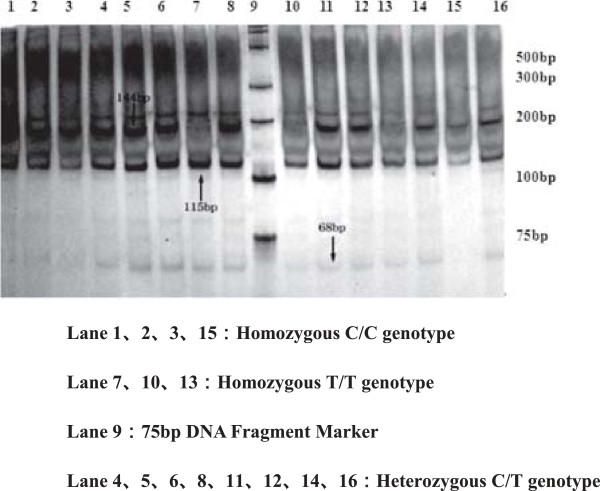
**Genotyping of the *SHMT C1420T *** **polymorphisms.**

**Figure 3:**
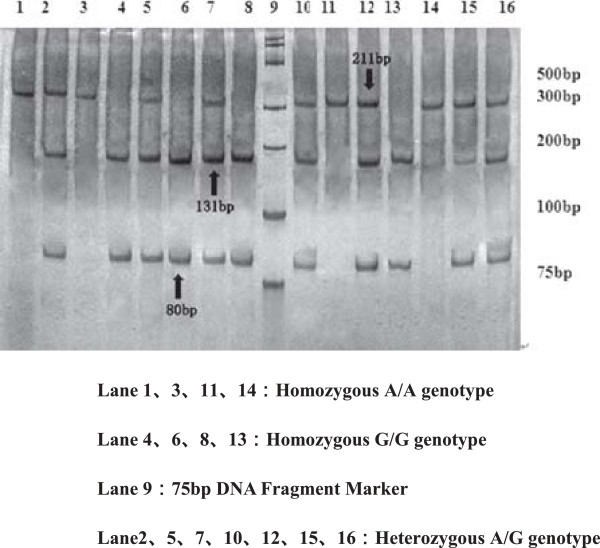
**Genotyping of the *MS A2756G *** **polymorphisms.**

**Figure 4:**
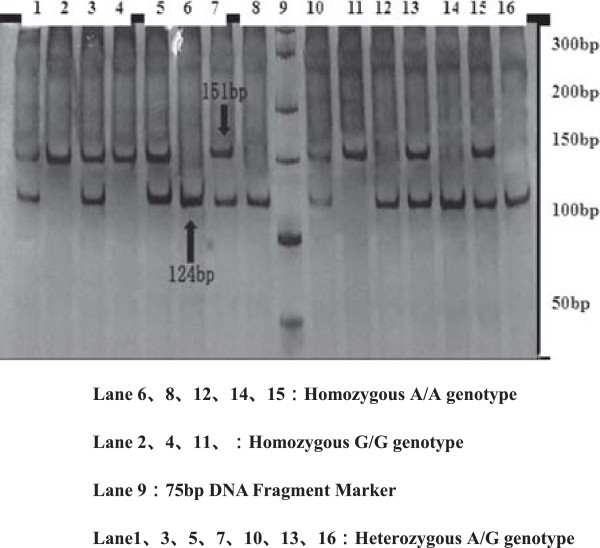
**Genotyping of the *MTRR A66G *** **polymorphisms.**

**Figure 5:**
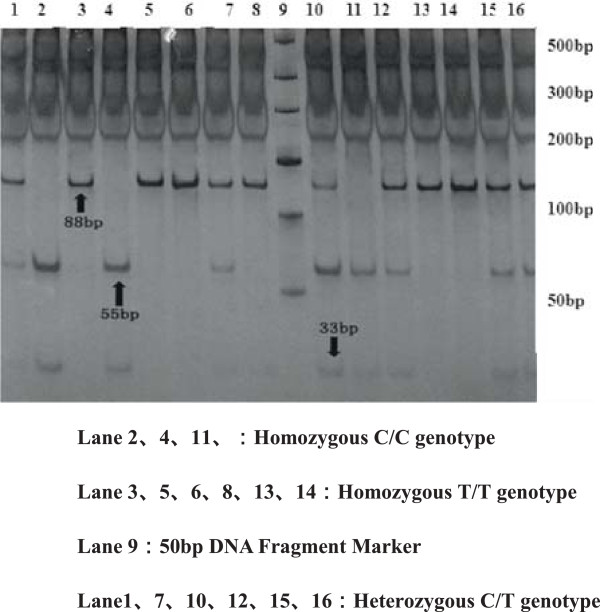
**Genotyping of the *CBS C699T *** **polymorphisms.**

**Figure 6:**
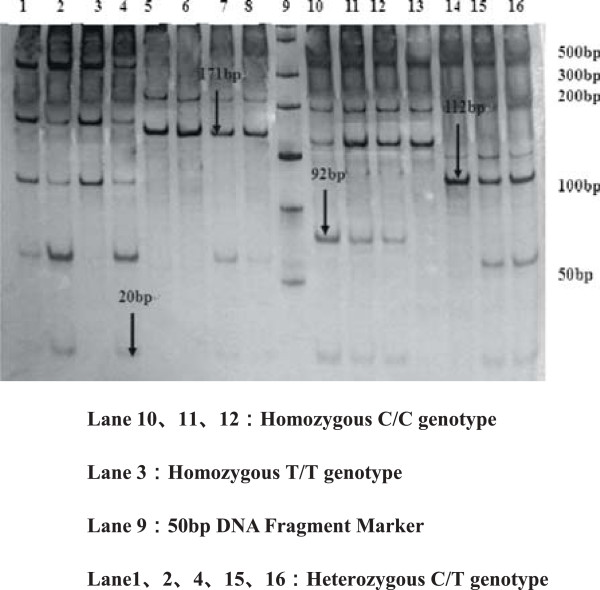
**Genotyping of the *CBS C1080T *** **polymorphisms.**

For polymorphisms of SHMT 1420T, MS 2756G, MTRR 66G allele are observed in Figures 2, 3, 4, 5 and 6, and result are shown in Tables 4 and 5 that *SHMT 1420CC, CT* and *TT* genotypes were 37.6%, 42.4%, 20%, C and T allele frequencies were 58.8%, 41.2% in control group, while *SHMT 1420CC, CT* and *TT* genotypes were 50%, 40.6%, 9.4%, C and T allele frequencies were 70.3%, 29.7% in case group. *SHMT C1420T* polymorphism of T allele frequency in case group was significantly lower than that in control group (p = 0.039). *SHMT 1420CT* and *SHMT 1420TT* genotypes can reduce the risk of breast cancer by 1.25 times and 2.5 times compared with *SHMT 1420CC*. The odds ratio between *SHMT 1420CT/TT*/*CT + TT* and breast cancer risk was observed to be 0.828 (95% CI = 0.54 ~ 1.27, p = 0.388) *vs.* 0.422 (95% CI = 0.40 ~ 1.71, p = 0.021) *vs.* 0.527 (95% CI = 0.55 ~ 1.24, p = 0.036) Figures 2, 3, 4, 5 and 6.

**Table 4:** **Genotype frequencies, allele frequencies at *SHMT C1420T,CBS C699T,CBS C1080T,MSA2756G,MTRR A66G *** **candidate loci**

***Genotype***	**Cases (n = 96) n (%)**	**Controls (n = 85) n (%)**	**Mutation Allelic frequency**		***p***^**a**^
			**Cases (%)**	**Controls (%)**	
*SHMT C1420T*	96(100.0)	85(100.0)	29.7	41.2	0.039^*^
*CC*	48(50.0)	32(37.6)			
*CT*	39(40.6)	36(42.4)			
*TT*	9(9.4)	17(20)			
*CBS C699T*	96(100.0)	85(100.0)	37.5	35.9	0.962
*CC*	38(39.6)	36(42.4)			
*CT*	44(45.8)	37(43.5)			
*TT*	14(14.6)	12(14.1)			
*CBS C1080T*	96(100.0)	85(100.0)	32.3	37.6	0.624
*CC*	47(49.0)	34(40.0)			
*CT*	36(37.5)	38(44.7)			
*TT*	13(13.5)	13(15.3)			
*MS A2756G*	96(100.0)	85(100.0)	20.3	11.1	0.004^**^
*AA*	59(61.4)	69(81.2)			
*AG*	35(36.4)	13(15.3)			
*GG*	2(2.2)	3(3.5)			
*MTRR A66G*	96(100.0)	85(100.0)	45.3	25.9	0.007^**^
*AA*	13(13.5)	9(10.6)			
*AG*	79(82.3)	75(88.2)			
*GG*	4(0.04)	1(0.12)			

^a^*Different from reference (p < 0.05), **different from reference (p < 0.01).

**Table 5:** **Odd rations (OR) and 95% CI for *SHMT C1420T,CBS C699T,CBS C1080T,MS A2756G and MTRR A66G *** **polymorphisms with risk of breast cancer**

**Genotype**	**Cases n (%)**	**Controls n (%)**	**OR**^**a **^**(95% CI**^**a**^**)**	***p***^**b**^
*SHMT 1420CC*	48(50.0)	32(37.6)	1.0 (Reference)	
*SHMT 1420CT*	39(40.6)	36(42.4)	0.828(0.54 to 1.27)	0.388
*SHMT 1420TT*	9(9.40)	17(20)	0.422(0.40 to 1.71)	0.021^*^
*SHMT 1420CT/TT*	48(50.0)	53(62.4)	0.527(0.55 to 1.24)	0.036^*^
*CBS 699CC*	38(39.6)	36(42.4)	1.0 (Reference)	
*CBS 699CT*	44(45.8)	37(43.5)	1.10(0.66 to 2.54)	0.69
*CBS 699TT*	14(14.6)	12(14.1)	1.14(0.38 to 1.83)	0.73
*CBS 699CT/TT*	58(60.4)	49(57.6)	1.27(0.45 to 1.65)	0.82
*CBS 1080CC*	47(49.0)	34(40.0)	1.0 (Reference)	
*CBS 1080CT*	36(37.5)	38(44.7)	1.0 (Reference)	0.86
*CBS 1080TT*	13(13.5)	13(15.3)	1.04(0.42 to 2.27)	0.90
*CBS 1080CT/TT*	49(51.0)	51(60)	1.15(0.69 to 3.11)	0.85
*MS 2756AA*	59(61.4)	61(71.8)	1.0 (Reference)	
*MS 2756AG*	35(36.4)	21(24.7)	2.49(0.29 to 0.833)	0.009^**^
*MS 2756GG*	2(2.2)	3(3.5)	1.72(0.13 to 2.11)	0.36
*MS 2756AG/GG*	37(38.5)	24(28.2)	2.32(0.29 to 0.82)	0.006^**^
*MTRR 66AA*	13(13.5)	9(10.6)	1.0 (Reference)	
*MTRR 66AG*	79(82.3)	75(88.2)	1.17(0.26 to 2.23)	0.51
*MTRR 66GG*	4(4.17)	1(1.18)	2.61(0.30 to 1.18)	0.03^*^
*MTRR 66AG/GG*	83(86.5)	76(89.4)	1.84(0.25 to 1.66)	0.047^*^

^a^OR, odd ratio; CI, confidence interval.

^a^Adjustment for age at enrollment and duration of fasting.

^b^*different from reference (p < 0.05), **different from reference (p < 0.01).

*MS 2756AA, AG* and *GG* genotypes were 81.2%, 15.3%, 3.5%, A and G allele frequencies were 88.9%, 11.1% in control group, while *MS 2756AA, AG* and *GG* genotypes were 61.4%, 36.4%, 2.2%, A and G allele frequencies were 79.7%, 20.3% in case group. *MS A2756G* polymorphism of G allele frequency in case group was significantly higher than that in control group (p = 0.004). The odds ratio between *MS 2756AG/GG*/*AG + GG* and breast cancer risk was observed to be 2.49 (95% CI = 0.29 ~ 0.833, p = 0.009) *vs.* 1.72 (95% CI = 0.13 ~ 2.11, p = 0.36) *vs.* 2.32 (95% CI = 0.29 ~ 0.82, p = 0.006).

*MTRR 66AA, AG* and *GG* genotypes were 10.6%, 88.2%, 1.2%, A and G allele frequencies were 74.1%, 25.9% in control group, while *MTRR 66AA, AG* and *GG* genotypes were 13.5%, 82.3%, 4.2%, A and G allele frequencies were 54.7%, 45.3% in case group. *MTRR A66G* polymorphism of G allele frequency in case group was significantly higher than that in control group (p = 0.007). The odds ratio between *MTRR 66AG/GG*/ *AG + GG* and breast cancer risk was observed to be 1.17 (95% CI = 0.26 ~ 2.23, p = 0.51) *vs.* 2.61 (95% CI = 0.30 ~ 1.18, p = 0.03) *vs.* 1.84 (95% CI = 0.25 ~ 1.66, p = 0.047) (Tables 3 and 4).

*CBS 1080CC, CT* and *TT* genotypes were 42.4%, 43.5%, 14.1%, C and T allele frequencies were 64.1%, 35.9% in control group, while *CBS 1080CC, CT* and *TT* genotypes were 49%, 37.5%, 13.5%, C and T allele frequencies were 67.7%, 32.3% in case group. There was no significantly different in *CBS C1080T* polymorphism of T allele frequency between case and control group. The odds ratio between *CBS 1080CT/TT*/ *CT + TT* and breast cancer risk was observed to be 1.31 (95% CI = 0.21 ~ 1.80, p = 0.86) *vs.* 1.04 (95% CI = 0.42 ~ 2.27, p = 0.90) *vs.* 1.15 (95% CI = 0.69 ~ 3.11, p = 0.85).

*CBS 699CC, CT* and *TT* genotypes were 40.0%, 44.7%, 15.3%, C and T allele frequencies were 62.4%, 37.6% in control group, while *CBS 699CC, CT* and *TT* genotypes were 49.0%, 37.5%, 13.5%, C and T allele frequencies were 67.7%, 32.3% in case group. There was no significantly different in *CBS C699T* polymorphism of T allele frequency between case and control group. The odds ratio between *SHMT 1420CT/TT*/*CT + TT* and breast cancer risk was observed to be 1.27 (95% CI = 0.66 ~ 2.54, p = 0.69) *vs.* 1.14 (95% CI = 0.38 ~ 1.83, p = 0.73) *vs.* 1.20 (95% CI = 0.45 ~ 1.65, p = 0.82).

### Hcy levels in different gene polymorphisms

Table 6 shows the plasma Hcy level of *SHMT 1420TT* homozygous were significantly lower than those of wild type *CC* in cases and controls [(14.86 ± 6.06) *vs.* (16.44 ± 17.69) μ mol/L in cases, p < 0.05; (10.02 ± 2.97) *vs.* (14.99 ± 7.12 μ mol/L in controls, p < 0.05]; plasma Hcy level of *MS 2756GG* genotype was significantly higher than that of wild type *AA* in cases and controls [(21.15 ± 23.30) *vs.* (12.23 ± 5.16) μ mol/L in cases, p < 0.001; (19.97 ± 12.88) *vs.* (10.71 ± 3.30) μ mol/L in controls, p < 0.01]; plasma Hcy level of *MTRR 66GG* homozygote genotype was significantly higher than that of wild type *AA* in cases [(23.70 ± 23.51) *vs.* (17.52 ± 5.69) μ mol/L, p < 0.05], Due to GG gene was only one in controls, there was no significant difference between three kinds of genotypes; plasma Hcy levels of *CBS 699TT* genotype was significantly higher than of wild type *CC* in cases and controls (19.52 ± 15.89) *vs.* (14.47 ± 3.32) μ mol/L in cases, p < 0.01; (15.47 ± 6.82) *vs.* (10.36 ± 5.12) μ mol/L in controls, p < 0.01]; plasma Hcy levels of *CBS 1080TT* genotype was significantly higher than that of wild type *CC* in cases and controls [(20.99 ± 16.89) *vs.* (15.76 ± 7.17) μ mol/L in cases, p < 0.01; (16.45 ± 9.45) *vs.* (9.28 ± 3.30) μ mol/L in controls, p < 0.001].

**Table 6:** **The effect of *SHMT C1420T,CBS C699T,CBS C1080T,MSA2756G,MTRR A66G *** **polymorphic genotype on Hcy concentration**

**Genotype**	**Plasma total Hcy(μ mol/L)**^**a**^		***p***^**b**^
	**Cases**	**Controls**	
*SHMT 1420CC*	16.44 ± 17.69	14.99 ± 7.12	0.15
*SHMT 1420CT*	18.74 ± 12.32	11.04 ± 4.64	0.01^**^
*SHMT 1420TT*	14.86 ± 6.06	10.02 ± 2.97	0.014^*^
	p = 0.021^*^(*TT&CC*)	p = 0.033^*^(*TT&CC*)	
*CBS 699CC*	14.47 ± 3.32	10.36 ± 5.12	0.01^**^
*CBS 699CT*	21.62 ± 13.08	11.20 ± 3.56	0.011^*^
*CBS 699TT*	19.52 ± 15.89	15.47 ± 6.82	0.019^*^
	p = 0.01^**^(*TT&CC*)	p = 0.028^**^(*TT&CC*)	
*CBS 1080CC*	15.76 ± 7.17	9.28 ± 3.30	0.01^**^
*CBS 1080CT*	16.64 ± 10.95	11.67 ± 2.36	0.021^*^
*CBS 1080TT*	20.99 ± 16.89	16.45 ± 9.45	0.01^**^
	p = 0.01^**^(*TT&CC*)	p = 0.01^**^(*TT&CC*)	
*MS 2756AA*	12.23 ± 5.16	10.71 ± 3.30	0.121
*MS 2756AG*	20.43 ± 15.32	13.39 ± 7.91	0.01^**^
*MS 2756GG*	21.15 ± 23.30	19.97 ± 12.88	0.042^*^
	p = 0.01^**^(*GG&AA*)	p = 0.002^**^(*GG&AA*)	
*MTRR 66AA*	17.52 ± 5.69	10.88 ± 2.67	0.01^**^
*MTRR 66AG*	15.83 ± 10.99	12.67 ± 5.13	0.040^*^
*MTRR 66GG*	23.70 ± 23.51	22.20	
	p = 0.031^*^(*GG&AA*)		

^a^Values are expressed as mean ± SD.

^b^*p < 0.05, **p < 0.01 compared with wild genotype.

### Comparison of plasma Hcy levels between case and controls with identical genotype

As shown in Table 6, plasma Hcy levels of *SHMT 1420CT/TT*, *CBS 699CC/CT/TT*, *CBS 1080CC/CT/TT*, *MS 2756AG/GG*, *MTRR 66AG/GG* group in cases were significantly higher than those in the controls (p < 0.001 ~ 0.01).

The results of the present study of associations between one-carbon metabolism-related gene polymorphisms or plasma Hcy levels and breast cancer risk suggested that (i) *SHMT 1420TT* genotype is inversely associated with breast cancer risk; (ii) the *MS 2756GG* or *MTRR A66G* genotype is positively associated with breast cancer risk; (iii) *SHMT 1420CC, MS 2756GG, MTRR 66GG, CBS 1080TT, CBS 699TT* genotype may be a factor in reducing plasma Hcy levels, and (iv) plasma Hcy level is positively associated with breast cancer risk.

Many observational studies have highlighted the importance of adequate folate intake in breast cancer prevention, as reviewed in a recent meta-analysis, but the results were inconsistent [34]. Almost all studies were conducted in predominantly USA populations, where fortification of folic acid intake with supplements causes difficulty in the evaluation of folate consumption [35]. On the other hand, folate intake in China is almost exclusively from natural sources, mainly from plant sources such as vegetables, with spinach making the highest contribution followed by rice and green tea [36]. However, few studies in Asia including China have investigated associations between folate intake and breast cancer risk [37] and further investigations in various populations are clearly warranted.

The folate metabolism pathway is complex and involves various enzymes that regulate DNA synthesis and DNA methylation. Among others, SHMT catalyzes the reversible conversion of serine and tetrahydrofolate to glycine and methylene tetrahydrofolate; MS supports methionine homeostasis by catalyzing the remethylation of homocysteine to methionine in a cobalamine-dependent reaction that utilizes 5,10-methylenetetrahydrofolate reductase (MTHFR) as methyl donor [26,38]; and MTRR is responsible for keeping MS in an active form by maintaining adequate levels of activated cobalamin, the enzyme cofactor for MS. Polymorphisms in the genes that encode these enzymes may modify the effect of folate on breast cancer.

Our results showed individuals with the *SHMT 1420TT* genotype were lower at risk of breast cancer, consistent with previous studies [39,40]. This is reasonable because enzyme activity with the *SHMT 1420CC* genotype is lower and thus less folate is available for DNA methylation. Our study also showed a significant interaction between the *MS A2756G* or *MTRR A66G* and risk of breast cancer. The *MS 2756GG* and *MTRR 66GG* genotype increases the risk among Chinese women in the present study. While functional effects have yet to be fully established, the G allele of MS and *MTRR* are considered to decrease the enzyme activity compared with the A allele [41,42]. Subjects with *MS 2756GG* or *MTRR 66GG* may have reduced methionine levels compared with those who had other genotypes and therefore our finding of increased breast cancer risk is plausible. In our study, *SHMT 1420 CC* genotype carriers were found to have increased plasma Hcy levels compared to *TT* carriers. For the MS 2756 A → G polymorphism, decreased Hcy levels have been found for the *GG* genotype as compared to the *AA* genotype. For *MTRR A66G* elevated Hcy levels for carriers of the homozygote wild-type genotype (*AA*) as compared to other genotypes. However, the results in this study may have occurred by chance due to small size of *SHMT 1420TT*, *MS 2756 GG* and *MTRR GG* genotype and information on *SHMT*, *MS* and *MTRR* polymorphisms and breast cancer is scarce, thus further investigations are needed.

The gene CBS, located on chromosome 21q22.3, encodes an important enzyme involved in transulfuration of Hcy produced during methyl-group metabolism [43]. Notably, CBS deficiency causes increased plasma methionine levels and decreased cysteine levels [44] which in turn are known to correlate with homocystinuria, cardiovascular disease and hepatocellular carcinoma [45,46]. The transulfuration pathway links methionine metabolism to the biosynthesis of cellular redox controlling molecules such as cysteine, glutathione, and taurine [47]. Cysteine generated through the transulfuration pathway determines cellular redox-controlling molecule levels, such as glutathione and taurine protecting cells against reactive species-induced damage [48] of DNA through base and sugar modifications, base-free sites, DNA-protein crosslinks, and strand breaks [49,50]. Thus, downregulated expression of CBS may impair the production of glutathione thus facilitating tumorigenesis [51]. In addition, redox imbalance stimulates protein kinase and poly-(ADP ribosylation) pathways leading to inhibition of apoptosis and resulting in necrotic cell death, followed by inflammatory responses and tumor development [52]. There are some studies that have evaluated the association between malignant tumor susceptibility and polymorphisms of the CBS gene (844ins68) in colorectal cancer [53,54] but not in esophageal or gastric cancer [55]. In addition, CBS 844ins68 polymorphism is associated with decreased survival in head and neck squamous cell cancer [56]. In the present study, no association between the breast cancer patients and the CBS 699/1080 alleles or genotypes was identified. These results corroborated recent findings of a lack of association, especially for the *TT* genotype of the CBS gene, with increased cancer risk. Furthermore, our results showed *CBS C699T*/ *C1080T* polymorphisms can remarkably increase plasma Hcy level, suggested *CBS* 699 or 1080 mutation maybe make the loss of CBS expression lead to the accumulation of Hcy, which will be recycled to methionine by MS via the remethylation pathway [57]. As methionine acts as the source of methyl group donor for DNA methylation, its increase caused by loss of CBS expression may dysregulate DNA methylation.

There are evidence for a role of Hcy in pathogenesis of cardiovascular diseases [18], renal failure [21] pregnancy complication [22], psychiatric and neurodegenerative disorders [24]. The Association between Hcy and breast cancer risk is unclear. In our study, we observed higher plasma Hcy levels in the breast cancer group, in accordance with some observation [25,58]. It was also shown that genetic variation in folate metabolism and inceased plasma Hcy levels was associated with an increased risk of breast cancer. Furthermore, a similar pattern of enhanced risk of breast cancer at higher plasma Hcy levels was observed in both pre-menopausal and post-menopausal women. This study seems to support the previously suggested role of Hcy as a potential tumor marker [43]. Hcy itself is toxic to human cells via its highly reactive lactone form which is incorporated into protein by methionyl-tRNA synthase [16]. Oxidation of thiol-containing amino acids produces free radicals having damaging effects on DNA [14], and their prooxidant effects have been implicated in DNA damage [21]. Decreased glutathione levels in some study indicate increased detoxification against oxidative stress in carcinogenesis. Indeed, a hypothesis for hyperhomocysteinemia as a risk factor for estrogen induced tumorigenesis has been proposed by Zhu [59]. Namely, elevated concentrations of Hcy exert pathogenic effects largely through metabolic accumulation of intracellular SAH, a strong non-competitive inhibitor of the COMT-mediated methylation metabolism of endogenous and exogenous catechols (including 2-OH-E2 and 4-OH-E2) [60]. It is well-known that the oxidative metabolites of estrogens, including the catechol estrogens (2-OH-E2/E1 and 4-OH-E2/E1) and 16a-OHE1, contribute to estrogen-induced tumors in certain animal models and to the development of human breast cancer [20,34,46]. The principal pathway for inactivation of catechol estrogens is O-methylation by COMT [22]. Accordingly, determination of Hcy levels is also important for protection from its toxic effects and for choosing therapy. Because Hcy is converted to SAH which is a potent inhibitor of DNA methyltransferase, so DNA methyltransferase activity and DNA methylation status should be taken into consideration in future.

## Conclusions

In summary, we have shown a significant association among *MS, MTRR, SHMT, CBS* polymorphisms, elevated plasma Hcy levels and increased risk of breast cancer. More research is needed regarding the functional effects of polymorphisms in these genes as related to effects on folate metabolism and subsequent folate availability and large populations are required to examine these gene-gene and gene-nutrient interactions using traditional epidemiologic methods. New methods of mathematical modeling developed for this pathway may provide insight into the effects of modifying components of the system to inform future studies. Additional studies are needed to replicate our findings in different racial/ ethnic groups and improve our ability to predict the effects of polymorphisms within genes in one-carbon metabolism and folate status. Future studies are needed to prove causality and provide insight on the mechanism of action of Hcy in breast tumorigenesis. In the study, there is big difference between the minimum age of cases (30y) and controls (18y) due to unequal number of cases and controls in order to ensure the average age is similar. The sample size was small and therefore the associations obtained need to be verified by prospective follow-up studies including a replication or larger number of subjects in future.

## Abbreviations

SHMT: Serine hydroxymethyhransferase; MS: Methionine synthase; MTRR: Methionine synthase reductase; Hcy: Homocysteine; CBS: Cystathionine beta synthase; MTHFR: 5,10-methylenetetrahydrofolate reductase; OR: Odds ratio; CI: Confidence interval; FPIA: Fluorescence polarization immunoassay; RFLP: Restriciton fragment length polymorphism.

## Competing interests

The authors declare that they have no competing interests.

## Authors’ contributions

XW is responsible the majority in this work, including execution of experiments, data analysis, composition of this report and publication; TZ is responsible for providing samples; NC and JN contributed some of the work and provided instruction; WX provided some of data analysis; TZ is responsible for proofreading work; XW as the corresponding author is responsible for the general work including experiments and publication. All authors have read and approved the final manuscript.
